# Perinatal mortality in rural Burkina Faso: a prospective community-based cohort study

**DOI:** 10.1186/1471-2393-10-45

**Published:** 2010-08-17

**Authors:** Abdoulaye Hama Diallo, Nicolas Meda, Emmanuel Zabsonré, Halvor Sommerfelt, Simon Cousens, Thorkild Tylleskär

**Affiliations:** 1 Centre MURAZ Research Institute, Ministry of Health/Burkina Faso, PO Box 390, Bobo-Dioulasso, Burkina Faso; 2 Centre for International Health (CIH), University of Bergen, Bergen, Norway; 3 Division of Infectious Diseases Control, Norwegian Institute of Public Health, Oslo, Norway; 4 London School of Hygiene and Tropical Medicine, Infectious Diseases Epidemiology Unit (IDEU), London, UK

## Abstract

**Background:**

There is a scarcity of reliable data on perinatal mortality (PNM) in Sub-Saharan Africa. The PROMISE-EBF trial, during which we promoted exclusive breastfeeding, gave us the opportunity to describe the epidemiology of PNM in Banfora Health District, South-West in Burkina Faso.

**Study objectives:**

To measure the perinatal mortality rate (PNMR) in the PROMISE-EBF cohort in Banfora Health District and to identify potential risk factors for perinatal death.

**Methods:**

We used data collected prospectively during the PROMISE-EBF-trial to estimate the stillbirth rate (SBR) and early neonatal mortality rate (ENMR). We used binomial regression with generalized estimating equations to identify potential risk factors for perinatal death.

**Results:**

895 pregnant women were enrolled for data collection in the EBF trial and followed-up to 7 days after birth. The PNMR, the SBR and the ENMR, were 79 per 1000 (95% CI: 59-99), 54 per 1000 (95% CI: 38-69) and 27 per 1000 (95% CI: 9-44), respectively. In a multivariable analysis, nulliparous women (RR = 2.90, 95% CI: 1.6-5.0), primiparae mothers (RR = 2.20, 95% CI: 1.2-3.9), twins (RR = 4.0, 95% CI: 2.3-6.9) and giving birth during the dry season (RR = 2.1 95% CI: 1.3-3.3) were factors associated with increased risk of perinatal death. There was no evidence that risk of perinatal death differed between deliveries at home and at a health centre

**Conclusion:**

Our study observed the highest PNMR ever reported in Burkina. There is an urgent need for sustainable interventions to improve maternal and newborn health in the country.

## Background

Reducing perinatal mortality, the sum of all stillbirths and early neonatal deaths (END), i.e. deaths within the first 7 days of life, is a major public health challenge in the low-income countries [1]. The annual global number of perinatal deaths was estimated by WHO to be 5.8 million in 2004 with 3 million stillbirths and 2.8 million ENDs [2].

Sub-Saharan Africa has the highest perinatal mortality rate (PNMR), estimated to be 56 per 1000 births in 2004, followed very closely by the Asian region with 47 per 1000 births [2]. Within the African region there is considerable variation, with the Central and West African regions having the highest perinatal mortality rates in the world, at 74 and 69 per 1000 births, respectively [2].

There is a paucity of reliable data on perinatal mortality in sub-Saharan Africa. This renders the problem invisible to donors and policymakers [3]. The need for better estimates of perinatal mortality in low-income countries, especially in Sub-Saharan Africa, has been stressed by several authors [2,4]. Such estimates are critical for the design and implementation of effective health programmes.

Burkina Faso is one of the least developed countries in the world with few and incomplete health statistics [5]. Recent estimates of the PNMR from the Demographic and Health Surveys (DHS) conducted in Burkina Faso range from 54 per 1000 in 1999 [6] to 36 per 1000 in 2003 [7].

We took the opportunity of the PROMISE-EBF study, during which we promoted exclusive breastfeeding counselling in 4 African countries [8,9], to study the epidemiology of perinatal mortality in a rural area of Burkina Faso. Our objectives were to provide estimates of the stillbirth rate (SBR) and the early neonatal mortality rate (ENMR) and to identify factors associated with the risk of perinatal death.

## Methods

### Study design

As part of the PROMISE-EBF trial, 24 villages in the Banfora Health District in the south-west of Burkina Faso were selected. All pregnant women enrolled in the 24 villages formed a prospective cohort that was followed-up until 7 days after birth.

### Study site

Banfora Health District covers an area of 15,000 Km^2 ^and a population of 385,000. The district is divided into three administrative sub-districts: Banfora, Soubakénédougou and Sidéradougou. Farming and animal husbandry are the main sources of income in all three sub-districts. The health care system consists of 60 primary health facilities (CSPS) and one regional hospital based in the town of Banfora [10].

### Participants' recruitment and follow-up

In each study village female "recruiters" were selected and trained to identify all pregnant women through weekly household visits and to record information on pregnancy outcomes. On average, each recruiter covered a population of 600. A list of newly identified pregnant women was prepared monthly and from this list up to four women were randomly selected from each village and asked to participate in the study. Recruitment of pregnant women lasted one year from June 2006 to May 2007.

Women selected for inclusion were visited by trained data collectors to confirm their eligibility and to seek written informed consent. They were eligible for inclusion in the study if they planned to live in the village for the next 12 months, were 7 or more months pregnant, intended to breastfeed their child, had no severe illness that would prevent breastfeeding and had no known mental handicap or psychiatric disorder.

Study participants were requested to inform the "recruiters" as soon as they had given birth, irrespective of the pregnancy outcome. Home visits were performed by data collectors at recruitment and at 7 days after birth for each mother-infant pair.

### Data collection and main variables measured

Under the guidance of two field supervisors, 5 data collectors who spoke at least two of the main local dialects (Dioula and Gouin/Karaboro), recorded data on handheld computers (PDA) using electronic questionnaires http://www.openXdata.org. They recorded information on the mother's age, parity, matrimonial status, household assets, medical history, use of health care services, the circumstances of the birth and the feeding pattern of the newborn. Mothers' height and newborns' birth weight were recorded from antenatal care (ANC) card.

A verbal autopsy was administered in the case of a stillbirth or infant death. We used the standard WHO definitions [1,11] of stillbirth and early neonatal death (END) to identify these two outcomes.

### Sample size of the EBF-trial

The sample size for the EBF trial was chosen in order to be able to detect a reduction in the prevalence of diarrheal morbidity at 12 weeks of age from 12% to 8% with a power of 90%. Assuming an average 35 infants per cluster, and a coefficient of variation between clusters of 0.3, we estimated that we needed 48 clusters per arm, 24 per country (12 in each arm).

### Data analyses

Data analysis was performed using Stata 10.1 (StataCorp, Texas 77845 USA). We analyzed categorical variables using Pearson chi-squared tests correcting for the cluster sampling design of the EBF-trial.

We generated a relative wealth index as a proxy for socio-economic status based on data collected at recruitment on the housing (construction material and roof), and household assets such as possession of the following items: car/truck, motorcycle/scooter, bicycle, mobile phone/telephone, plough and chart. The index was constructed using principal component analysis [12,13]. Classes were derived by dividing the index into quintiles.

Evidence that the PNMR, SBR and ENMR varied across clusters was sought by fitting a logistic regression model with cluster as a random-effect.

Binomial regression with generalized estimating equations (GEE) to adjust for village clustering was used to obtain risk ratio (RR) estimates and their 95% confidence intervals (CIs). Groups with the lowest PNMR were used as reference category for each variable. Because the number of ENDs was small and there is the potential for misclassification between stillbirths and ENDs, we present only an analysis of risk factors for perinatal death including all births (n = 915).

Multivariable regression analyses included both the variables associated with risk of perinatal death (P < 0.10) in the univariable analysis, and those previously reported to be associated with perinatal death. All multivariable models accounted for the cluster-design using GEE.

### Ethical and administrative clearances

The study was approved by the Institutional Review Board of Centre MURAZ (N°013/2005/CE-CM) and the Western Regional Committee for Medical and Health Research Ethics in Norway (Sak nr. 05/8197). The PROMISE-EBF-trial is registered at http://www.clinicaltrials.gov under NCT00397150. The study was authorized by the Ministry of Health of Burkina Faso. Medications for fever, malaria, acute respiratory infections, diarrhoea, and breast abscesses were made available in all primary health facilities in Banfora Health District and provided free of charge to all the study participants throughout the duration of the PROMISE-EBF-trial.

## Results

### Study profile

A total of 1162 pregnant women were identified. Twenty-one women (1.8%) declined study participation at the initial contact with recruiters. Nine hundred women were randomly selected for follow-up of whom 895 were eligible, all of whom were followed-up until 7 days after delivery. Of the five women excluded, 3 had given birth before they could be enrolled, one woman was found to have a mental handicap and another was not actually pregnant. At birth, there were 20 pairs of twins, so the total number of fetuses at risk of perinatal death was of 915.

### Characteristics of mothers

The mean age of women at recruitment was 26 years, with most aged between 20 and 35 years (Table 1). Only 17% of the women were nulliparous. Most of the women (>80%) had never been to school. Nearly 10% of multiparous mothers had experienced a previous perinatal death. The median gestational age at enrolment was 8 months. Of the 365 women with known height at inclusion, only two (0.5%) were below 150 cm. Six-hundred-and-forty-two women (72%) went for antenatal care (ANC) before delivery. Of these, 47% had their height and 55% had their fundal height recorded on the ANC card. ANC attendance varied with parity and 22% of nulliparous mothers completed more than 2 ANC visits compared to 17% among multiparous mothers (p = 0.01). There was no evidence that ANC attendance varied with distance to the nearest health facility (p = 0.36), season of the year (p = 0.55), maternal education (p = 0.42) or socioeconomic status as assessed by household assets (p = 0.35).

**Table 1:** Maternal characteristics and unadjusted risk ratio (RR) for perinatal death among 895 pregnant women enrolled in the PROMISE-EBF study in Banfora Health District.

Variables	Number (%) n = 895	Perinatal deaths (N = 72)	Number of births n = 915	PNMR Per 1000 births	**Unadjusted RR**^**†**^ [95% CI]
Area					
- Banfora	440 (49.0)	41	451	90.9	1.36 [0.7-2.4]
- Soubakénédougou	221 (25.0)	15	225	66.7	1
- Sidéradougou	234 (26.0)	16	239	66.9	1.0 [0.5-2.0]
Maternal age					
- <20	148 (16.5)	22	149	147.7	2.71 [1.0-6.9]
- 20-35	656 (73.3)	45	674	66.8	1.22 [0.5-3.0]
- >35	91 (10.2)	5	92	54.3	1
Parity					
- 0	153 (17.0)	19	154	123.4	2.19 [1.2-3.9]
- 1	146 (16.3)	15	150	100.0	1.78 [0.9-3.3]
- 2-4	412 (46.0)	24	425	56.5	1
- ≥5	184 (20.7)	14	186	75.3	1.33 [0.7-2.5]
Educational level					
- None	720 (80.4)	58	736	78.8	1.05 [0.4-2.8]
- Literacy/primary school	120 (13.4)	10	124	80.6	1.08 [0.3-3.3]
- Secondary school	55 (06.2)	4	55	72.7	1
Use of media (radio, TV)					
- Everyday	166 (18.6)	9	169	53.3	1
- Rare/None	729 (81.4)	63	746	84.5	1.58 [0.8-3.1]
Assets ownership (household)					
- Quintile 1 (most poor)	191 (21.0)	15	193	77.7	1.10 [0.4-2.9]
- Quintile 2	168 (19.0)	22	175	125.7	1.78 [0.7-4.5]
- Quintile 3	189 (21.0)	13	189	68.8	0.92 [0.3-2.5]
- Quintile 4	172 (19.0)	9	180	50.0	0.87 [0.3-2.3]
- Quintile 5 (least poor)	175 (20.0)	13	178	73.0	1
Distance to nearest health facility					
- ≤5 km	429 (48.0)	39	442	88.2	1.26 [0.8-2.0]
- >5 Km	466 (52.0)	33	473	69.8	1
History of perinatal death^**‡**^					
- Yes	88 (09.8)	9	90	100.0	1.52 [0.8-3.0]
- No	654 (90.2)	63	671	65.6	1
Used bednet during pregnancy					
- Yes	341 (38.1)	26	349	74.5	1
- No	554 (61.9)	46	566	81.3	1.10 [0.7-1.8]
Antenatal care visits					
- None	253 (28.3)	23	256	89.8	1.52 [0.7-3.1]
- 1-2	481 (53.7)	39	492	79.3	1.33 [0.7-2.6]
- >2	161 (18.0)	10	167	60.0	1
Season of birth					
- Dry season (Nov-April)	431 (48.0)	47	440	106.8	2.02 [1.3-3.2]
- Rainy season (May-Oct)	464 (52.0)	25	475	52.6	1
Place of birth					
- Health facility	319 (36.0)	26	326	79.8	1.02 [0.6-1.6]
- Home/TBA's place/other	576 (64.0)	46	589	78.1	1
Birth attendants					
- Health personnel	335 (37.0)	26	342	76.0	1
- TBA/Family/none	560 (63.0)	46	573	80.3	1.06 [0.7-1.7]
Had twins'birth					
- Yes	20 (2.0)	8	40	200.0	2.78 [1.4-5.3]
- No	875 (98.0)	64	875	73.1	1

^**†**^Univariable analyses adjusted for clustering to account for the design of the PROMISE-EBF Trial

^**‡**^Restricted to multiparous mothers

The median time from enrolment to birth was 52 days (7.4 weeks), ranging from 0 to 119 days. About one third (36%) delivered in a health facility. Over half of the women (54%) gave birth in their own home, while 9% delivered in a traditional birth attendant's (TBA's) home. Nine women (1%) gave birth in other places (6 in the fields, 2 at the market place and one woman on her way to the health facility). Sixteen women (2.7%) who did not deliver in a health-facility were assisted by trained health personnel at birth.

The proportion of deliveries assisted by a skilled attendant (doctor, midwife, nurse) decreased with increasing distance of the village to the nearest primary health facility (from 53% in villages within 5 km of a facility, to 23% for villages over 5 km from a facility, p < 0.001). Health facility delivery was strongly correlated to the type of attendant at birth (Pearson's coefficient = 0.95, 95% CI: 93-97) and we observed a higher probability of facility delivery among nulliparous mothers (48%) compared to multiparous mothers (33%) with a RR = 1.25 (95% CI: 1.1-1.45).

Among those with live births (866 women), 17 (2%) reported a complicated labour, of whom 7 reported obstructed labour, 7 reported severe haemorrhage and 3 reported placental retention. Eight women (0.9%) underwent caesarean-section. Fifty-one percent of the live births were male. Only 293 babies had their birth weight measured, all of whom were born in health facilities; their mean birth weight was 2975 g with a standard deviation (SD) of 524 g. The prevalence of low birth weight (<2500 g) in this sample was of 13.6%. Breastfeeding was initiated within one hour by 3.6% of women, within 12 hours by 52.8% and within 24 hours by 83.2% of women with live births.

### Outcome of pregnancy

The 895 pregnancies resulted in 915 births, of which 49 were stillbirths (SBR = 53.6 per 1000 births, 95% CI: 38.3 to 68.8). There was no stillbirth among the twins. There were 23 ENDs (ENMR = 26.6 per 1000 live births, 95% CI: 9.4 to 43.7) of whom 8 were twins, and two maternal deaths (MMR = 231 per 100,000 live births, 95% CI: 28 to 832). Thus, the PNMR was 78.7 per 1000 births (95% CI: 58.8 to 98.6) with stillbirths accounting for 68% of all perinatal deaths.

### Distribution of stillbirths

Stillbirths occurred in 21 out of 24 villages. The highest observed risk in a village was 151.5 per 1000 births (Table 2). However, there was no evidence that the variation in observed risk of stillbirth across clusters was greater than might be expected by chance (LR test, p = 0.49).Thirty-one (67%) of stillbirths occurred at home. Emotional and cultural factors related to stillbirths in the Banfora region limited the data we were able to collect, so neither the gender of the fetal losses, nor the complications at birth were recorded for this group of mothers.

**Table 2:** Distribution of stillbirth rate (SBR), early neonatal mortality rate (ENMR) and perinatal mortality rate (PNMR) per village (cluster) among 915 births in Banfora Health District (Burkina Faso)

No	Village	Number of births	SBR N (/1000)	ENMR N (/1000)	PNMR N (/1000)
1	Nafona1	38	2 (52.6)	6 (166.7)	8 (210.5)
2	Karfiguéla	29	1 (34.5)	4 (142.9)	5 (172.4)
3	Noumousso	33	5 (151.5)	0 (0)	5 (151.5)
4	Zédougou	51	6 (117.6)	0 (0)	6 (117.6)
5	Kotou	36	4 (111.1)	0 (0)	4 (111.1)
6	Sikanadjo	19	1 (52.6)	1 (55.6)	2 (105.3)
7	Tiempangora	38	3 (78.9)	1 (28.6)	4 (105.3)
8	Kirbina	31	3 (96.8)	0 (0)	3 (96.8)
9	Laferma	33	1 (30.3)	2 (62.5)	3 (90.9)
10	Siniéna	91	5 (54.9)	3 (34.9)	8 (87.9)
11	Lémourou.village	30	1 (33.3)	1 (34.5)	2 (66.7)
12	Tiékouna	30	2 (66.7)	0 (0)	2 (66.7)
13	Damana	45	3 (66.7)	0 (0)	3 (66.7)
14	Lémourou.Cité	16	0 (0)	1 (62.5)	1 (62.5)
15	Kouéré	52	2 (38.5)	1 (20.0)	3 (57.7)
16	Dègue-Dègue	37	2 (54.1)	0 (0)	2 (54.1)
17	Gouin-Gouin	40	1 (25.0)	1 (25.6)	2 (50.0)
18	Niamirandougou	40	1 (25.0)	1 (25.6)	2 (50.0)
19	Tangora	43	2 (46.5)	0 (0)	2 (46.5)
20	Létiéfesso	44	2 (45.5)	0 (0)	2 (45.5)
21	Gouindougouba	45	1 (22.2)	1 (22.7)	2 (44.4)
22	Kossara	23	1 (43.5)	0 (0)	1 (43.5)
23	Tatana	30	0 (0)	0 (0)	0 (0)
24	Boborola	41	0 (0)	0 (0)	0 (0)
	Mean (SD)*	38.1 (14.4)	52.0 (37.7)	28.4 (44.3)	79.3 (49.4)

* Mean (SD) obtained from cluster-level summaries of the data collapsed by village.

Cluster are ranked by decreasing order of PNMR, Nafona 1 and Karfiguéla both located in the Banfora's administrative area are the clusters with the highest PNMR.

### Distribution of early neonatal deaths (ENDs)

Twenty-three ENDs occurred in 12 of the 24 villages. There was evidence that ENMR varied between clusters (LR test, p = 0.002) with Karfiguéla and Nafona1 having the highest ENMR (Table 2). The median age at death for these neonates was 3 days and 26% died within 24 hours of birth. Twelve of the babies (52%) who died were born at home. Eleven (48%) were boys and only one baby had his birth weight available (3690 g). Twenty-one of the deaths (91%) occurred at home and only 4 of the babies (17%) were taken to a health facility prior to death. The probable causes of death based on the verbal autopsy information were: complications of preterm birth (N = 11), hemorrhage (N = 1), birth asphyxia (N = 1) and infection (N = 3). The cause was unknown for 7 babies.

### Factors associated with perinatal death

There was no evidence of an association between the distance to the nearest health facility and the risk of perinatal death (Table 1).

The unadjusted analysis showed that nulliparous mothers (RR = 2.19, 95% CI: 1.2 to 3.9), a birth in the dry season (RR = 2.02, 95% CI: 1.3 to 3.2), and twinship (RR = 2.78, 95% CI: 1.4 to 5.3) were factors associated with increased risk of perinatal death (Table 1). There was a weak evidence of an association between perinatal death and primiparae mothers, and also with young age of the mother. Neither the place of birth, nor the type of birth attendant, nor any of the other factors examined showed evidence of an association with the perinatal death risk (Table 1).

Multivariable model 1 that accounted for clustering and included 6 explanatory variables (Table 3) confirmed the results of the crude analysis. Twins were 4 times higher risk of perinatal death than singletons (RR = 4.0, 95% CI: 2.3 to 6.9). Nulliparous and primiparae mothers had increased risk of perinatal death with RRs of 2.9 (95% CI: 1.6 to 5.0) and 2.2 (95% CI: 1.2 to 3.9), respectively, compared to multigravidae with 2-4 previous births. Birth during the dry season remained associated with increased risk of perinatal death (RR = 2.1, 95% CI: 1.3 to 3.3).

**Table 3:** Multivariable regression analysis of the risk factors for perinatal death in a cohort of 915 births in Banfora Health District, Burkina Faso.

Variables	**Unadjusted**^**¤**^ RR [95% CI] n = 915	**Adjusted model 1**^**†**^ RR [95%CI] n = 915	**Adjusted model 2**^**§**^ RR [95%CI] n = 761
Parity			
- 0	2.19 [1.2-3.9]	2.90 [1.6-5.0]	
- 1	1.78 [0.9-3.3]	2.20 [1.2-3.9]	2.20 [1.2-3.9]
- 2-4	1	1	1
- ≥ 5	1.33 [0.7-2.5]	1.42 [0.7-2.7]	1.36 [0.7-2.6]
Use of media (radio, TV)			
- Everyday	1	1	1
- Rare/None	1.58 [0.8-3.1]	1.50 [0.7-2.9]	1.42 [0.6-3.2]
History of perinatal death^**‡**^			
- Yes	1.52 [0.8-3.0]		1.40 [0.7-2.8]
- No	1		1
Used bednet during pregnancy			
- Yes	1	1	1
- No	1.10 [0.7-1.8]	1.03 [0.7-1.6]	1.07 [0.6-1.8]
Antenatal care visits			
- None	1.52 [0.7-3.1]	1.50 [0.8-2.9]	1.54 [0.6-3.8]
- 1-2	1.33 [0.7-2.6]	1.21 [0.6-2.2]	1.30 [0.5-3.1]
- >2	1	1	1
Season of birth			
- Dry season (Nov-April)	2.02 [1.3-3.2]	2.10 [1.3-3.3]	2.46 [1.4-4.4]
- Rainy season (May-Oct)	1	1	1
Pair of twins			
- Yes	2.78 [1.4-5.3]	4.00 [2.3-6.9]	3.1 [1.5-6.2]
- No	1	1	1

^**¤**^Adjusted for clustering to account for the PROMISE-EBF study design.

^**†**^Adjusted for clustering and all variables in the table except the history of perinatal death.

^**§**^Adjusted for clustering and for all variables in the table

^**‡**^Includes only multiparous mothers

Overall, pauciparae women (nulliparous and primiparae), twins and a birth during the dry season were the factors significantly associated with the risk of perinatal death in this cohort.

Excluding nulliparous women (multivariable model 2) did not materially alter the results.

## Discussion

### Estimates of perinatal mortality

This study highlights the high perinatal mortality in a rural area of Burkina Faso. The PNMR of 78.7‰ (95% CI: 58.7.8-98.6) is to our knowledge the highest PMNR ever reported from Burkina Faso. The last DHS estimated the PNMR nationally at 35 per 1000 with 33 per 1000 in rural settings [7]. More recent studies conducted at community-level in Burkina Faso have reported PNMRs of 32 per 1000 in Houndé [14], 33 and 28 per 1000 in Diapaga and Ouargaye, respectively [15]. A study conducted in the rural district of Boromo showed also a high risk of fetal loss (48 per 1000) in a context of community-based promotion of intermittent preventive treatment with sulfadoxine-pyrimethamine [16].

We conducted a prospective, community-based, cohort study in which local "recruiters" were involved in identification and follow-up of pregnant women in each cluster. They recorded on a daily basis all the pregnancy outcomes and provided our study team with timely information on each study participant. In such a study, the number of events recorded is expected to be more accurate and may be higher than that identified in a survey using recall such as the DHS [7]. The few prospective studies conducted in similar settings in Burkina were either health-facility-based [17], used a different source of informants [15], or carried a higher likelihood of recall bias [18]. Our cohort, with a very low proportion of refusers (1.8%) was drawn from a random sample of pregnant women identified in each village and is accordingly less prone to selection bias.

The rural location of the study clusters may have contributed to the high PNMR. It is known that in rural settings in Burkina Faso, there is limited access to and utilization of health facilities [19,20]. In our study, only 25% of the clusters had a health facility in the village and the proportion of assisted delivery was low (37%). Where health facilities exist and are accessible, the quality of health care offered may be poor, due to under-staffing or demotivated health personnel [4,21]. In this study, the proportions of pregnant women with ANC visits who had either their height or their fundal height measured were low, and the proportion of deliveries done by caesarean section was very low, both an indication that there is a substantial scope of improving the quality of antenatal and childbirth services in rural areas of Burkina Faso.

The high ratio of stillbirths to early neonatal deaths (2.1:1) could be partially explained by misclassification. Accurate ascertainment of gestational age is difficult in populations such as this and some of the foetal losses recorded as stillbirths may have occurred before 28 weeks of gestation, resulting in an SBR overestimate. However all women included in this study claimed to be over 6 months of gestation at enrollment and the median time from inclusion to birth (7.4 weeks) is consistent with this.

A second source of misclassification in such a study, with a high proportion of home deliveries, is if some ENDs were reported by mothers as being stillborn. In our study, the stillbirths represented 68% of all perinatal deaths rather more than the 50-60% typically reported by other sub-Saharan Africa studies [22,23]. The reasons for such biased reporting may rest in socio-cultural factors as suggested by previous studies in sub-Saharan Africa [3,24].

The ENMR (26.6 per 1000 live births) is consistent with previous findings from DHS (21 per 1000 in 2003) in Burkina Faso. The probable causes identified among ENDs are consistent with those of others [3,4] except for the low proportion of deaths attributed to birth asphyxia, which may reflect misclassification of live babies not breathing at birth as stillbirths.

PNMRs similar to that in our study have been reported elsewhere in Africa. A recent study from the Democratic Republic of Congo reported a PNMR of 64 per 1000 [23], and two other African studies [25,26] reported PNMRs of 74.4 and 68.3 per 1000, in the Gambia and Malawi, respectively. The estimates provided in this study also fall within the range of WHO's estimates of PNMR for the African region which were estimated in 2004 at 69 and 74 per 1000 in the West and Central African region, respectively [2].

We believe that the weak health system in Burkina Faso [20,27] may have contributed substantially to this high PMNR in rural Burkina Faso. The fact that there was no evidence of risk difference between deliveries at home and at health facilities points in this direction. Emergency obstetric care that is a crucial component of any strategy aiming at the reduction of perinatal deaths is not readily accessible in rural settings [28].

### Risk factors for perinatal death

In our study, twinship, nulliparous and primiparae mothers, and giving birth during the dry season were the three factors most strongly associated with perinatal death risk. Previous studies have reported primigravidae and nulliparous women as groups at high risk for perinatal death in Africa [17,23,25,29]. Among the reasons listed for this is the higher risk of obstructed and complicated labour [30,31]. Obstructed labour often requires emergency obstetric care which is only available at the district hospital. Moreover, Banfora is a holoendemic malaria area, a disease reported to be associated with poor pregnancy outcomes especially among primigravidae and secundigravidae [32,33]. Intermittent preventive treatment (IPT_p_) with sulfadoxine-pyrimethamine to prevent placental malaria was implemented recently in Burkina Faso [34] but was not widely available at the time of our study.

The twinning rate in our study is consistent with that reported previously [6,23]. Twins have been reported to be at high risk of perinatal death in several studies [23,35] and our results confirm that the same pattern prevails in rural Burkina Faso. Pre-term birth and intra-uterine growth restriction are two factors suggested to increase the vulnerability of multiple births to perinatal death [23,36].

The seasonal pattern of perinatal mortality observed in our study has not previously been reported in Burkina Faso. Our findings are consistent with those reported in a recent Tanzanian study where the authors found that perinatal mortality was higher during the dry season [29]. In Burkina Faso, the few studies which looked at seasonal pattern of child mortality focused on a different study population, i.e. of children from 1-60 months of age, and perhaps unsurprisingly found a different pattern [37-39]. It is known that the causes of perinatal deaths differ from those of post-neonatal deaths [4,22]. We do not have an explanation for the seasonal pattern observed in our study. However, the observed pattern does not exclude the potential role of malaria as women delivering during the dry season may have been exposed to this parasitic infection on earlier stages of their pregnancy.

Our study is limited by its relatively small sample size, resulting in a low power to detect risk factors associated with small increases in perinatal death risk, by our inability to distinguish antepartum from intra-partum stillbirths and by the likely misclassification of some ENDs as stillbirths. Literature suggests that almost two third of stillbirths in resource-limited settings occur intrapartum and, as such, include avoidable deaths [1,30].

## Conclusion

Perinatal mortality is very high in this rural area of Burkina Faso. There is an urgent need for sustainable interventions to improve maternal and newborn health in the country. Nulliparous and primiparae mothers and twins are high risk groups while the seasonal pattern observed in our study needs further investigation.

## Competing interests

The authors declare that they have no competing interests.

## Authors' contributions

AHD, NM, HS and TT have designed the study. AHD conducted the study, performed data analysis and drafted the manuscript. EZ contributed to data collection and data analysis. NM, TT, HS, and SC contributed to data analysis. All authors read and approved the manuscript.

## The Promise-EBF study group*

List of members for the PROMISE-EBF Study Group

Steering Committee:

Thorkild Tylleskär, Philippe Van de Perre, Eva-Charlotte Ekström, Nicolas Meda, James K. Tumwine, Chipepo Kankasa, Debra Jackson.

Participating countries and investigators:

*Norway: *Thorkild Tylleskär, Ingunn MS Engebretsen, Lars Thore Fadnes, Eli Fjeld, Knut Fylkesnes, Jørn Klungsøyr, Anne Nordrehaug-Åstrøm, Øystein Evjen Olsen, Bjarne Robberstad, Halvor Sommerfelt

*France: *Philippe Van de Perre

*Sweden: *Eva-Charlotte Ekström, Barni Nor

*Burkina Faso: *Nicolas Meda, A. Hama Diallo, Thomas Ouedrago, Jeremi Rouamba, Bernadette Traoré, Germain Traoré, Emmanuel Zabsonré

*Uganda: *James K. Tumwine, Caleb Bwengye, Charles Karamagi, Victoria Nankabirwa, Jolly Nankunda, Grace Ndeezi, Margaret Wandera

*Zambia: *Chipepo Kankasa, Mary Katepa-Bwalya, Chafye Siuluta, Seter Siziya

*South Africa: *Debra Jackson, Mickey Chopra, Mark Colvin, Tanya Doherty, Ameena E Goga, Lungiswa Nkonki, David Sanders, Wesley Solomons Wanga Zembe.

(Country PI first, others in alphabetical order of surname)

## Sources of funding

The PROMISE-EBF study was funded by the European Commission Framework Programme-6 under the contract INCO-CT-2004-003660. The sponsor had no responsibility in the design, conduct, analysis, interpretation or publication of the data.

## Pre-publication history

The pre-publication history for this paper can be accessed here:

http://www.biomedcentral.com/1471-2393/10/45/prepub
